# Process evaluation of paediatric fellowship training programs at a University Hospital in Pakistan

**DOI:** 10.1186/s12909-023-04501-z

**Published:** 2023-08-28

**Authors:** Sana Saeed, Prem Chand, Asna Sulaiman, Imran Nisar, Khadija Nuzhat Humayun, Marib Ghulam Rasool Malik, Fyezah Jehan

**Affiliations:** Department of Paediatrics & Child Health, The Aga Khan University Hospital, Stadium Road, PO Box 3500, Karachi, 74800 Pakistan

**Keywords:** Program evaluation, Training programs, Fellowship programs, Quality

## Abstract

**Background:**

Fellowship programs offer training in a subspecialty focusing on distinct and advanced clinical/academic skills. This advanced postgraduate training allows physicians, who desire a more specialized practice, to further develop clinical, academic, research, and leadership/administrative skills. The Aga Khan University (AKU) is one of the few institutes offering paediatric sub-specialty training in Pakistan. We aimed to evaluate the current Paediatric fellowship programs at AKU.

**Methods:**

Process evaluation of six paediatric fellowship programs (cardiology, neurology, endocrinology, critical care medicine, neonatology, and infectious disease) was conducted from September 2020 to April 2021 by senior clinicians and medical educationists. Evidence was collected through document review (using existing postgraduate medical education program information form), observation of teaching and learning support, and focused group discussions/interviews with program faculty and fellows were conducted. A review of the evaluation report was done as part of this study. This study received an exemption from the ethical review committee. The quantitative data were analyzed using SPSS (22.0) while the reports of discussion with fellows and friends underwent content analysis.

**Results:**

All fellowship programs met the criteria for having a robust competency-based fellowship curriculum as per the institutional and national guidelines. Formative assessment in the form of continuous evaluation was found to be integrated into all the fellowship programs, however, most of the programs were found to lack a summative assessment plan. Fellows in training and program faculty were satisfied with the opportunities for mentorship, teaching, and learning. Thematic analysis of the discussion reports with faculty and fellows revealed three key themes including, program aspects translating into strengthening the training, gaps in the training program in delivering the expectations, and making ways to reach par excellence.

**Conclusions:**

The process evaluation of paediatric fellowship programs provided an opportunity to holistically review the current strengths and quality of the training in individual programs along with the unmet needs of the trainees. This will help the program stakeholders to prioritize, align and allocate the resources to further enhance the quality of training and outcome of individual fellowship programs to ensure wider impacts at a regional, national, and international health system level.

## Background

Fellowship is a training program, following core residency training, which focuses on the development of both clinical and non-clinical abilities at a subspecialist level [1]. This advanced postgraduate training allows physicians, who desire a more specialized practice, to further develop clinical, academic, research, and leadership/administrative skills [2]. In the current times, post-residency graduates are determined, more than ever, to attain further clinical competence, confidence, and specialized skills [3]. Hence, the institutions must rise to meet these growing demands. Tertiary care hospital settings the world over are known to set the benchmark for academics and training [4]. Globally, not only the number and proportion of residents pursuing fellowship training has rocketed but, the number of fellowship programs, in institutes, is also increasing [5]. Karpinski et al., reported that 60–85% of Canadian residency graduates intend to pursue a fellowship, with similar findings from studies carried out in the west [5–7]. This trend not only provides academic benefits with focused training for learners but also leads to the facilitation of research productivity and improvement in the volume and quality of clinical services through the specialist doctors competent to establish a safe healthcare system and to address the need of the community. Similarly, the College of Physician and Surgeons Pakistan (CPSP) reported progressive expansion of postgraduate training in the country from seven medical specialists in 1947 to eighteen thousand in 2007 [8].

Medical programs are based on a non-linear training structure where there is constant evolution based on community needs as well as national and international benchmarks of quality in training. The evaluation of program elements and outcomes contribute to its continuing development and quality improvement thereby bringing forth excellence in patient care [9]. Accreditation of the training programs involves validation of the work environment, academics, and its balance with service, evaluations, mentorship, and opportunities for quality supervision [10]. Periodic scientific evaluation of training programs has long been advocated to ensure a safe and productive environment for the trainees which in turn affects performance and adequate patient care [3, 11]. Globally, studies on the evaluation of fellowship programs are commonly conducted, with data available in the form of both cross-sectional surveys as well as process evaluations for medical, surgical, and paediatric subspecialty programs [2, 12, 13]. However, literature suggests that such studies, limited by the quality of their designs, often lack a robust mechanism to evaluate the overall performance of the programs or assess the individual impact of the specific aspects of the training [14].

The Aga Khan University (AKU) is one of the few institutes offering paediatric sub-specialty training programs in Cardiology, Critical Care Medicine, Endocrinology, Infectious Diseases, Neurology, and Neonatology in Karachi, Pakistan. These training programs are in alignment with the mission of AKU, which is, “to prepare individuals for constructive and exemplary leadership roles, and shaping public and private policies, through strength in research and excellence in education, all dedicated to providing meaningful contributions to society”. The first paediatric fellowship programme in neonatology was birthed in 1996 and soon got recognition by the CPSP. These fellowship programmes follow the policies and standardized training requirements as set by the Postgraduate Medical Education (PGME) -AKU and CPSP.

Although global data regarding evaluation of programs in different parts of the world is available, there is minimal literature describing the evaluation of fellowship training in our local context [15]. Through this process, we aimed to undertake a formative evaluation of current paediatric fellowship programs at AKU. Our goal was to improve the academic quality and training framework of the training programs to achieve outcomes of improving standards for international accreditation and subsequent growth of these programs which ultimately lead to improved patient care. We are presenting the process and results from this endeavor to add to the available body of literature on this subject.

## Methods

### Setting

The program audit took place from September 2020 to April 2021 at the Department of Paediatrics and Child Health, AKU. This study has been given an exemption from AKU- ethical review committee (ERC# 2021-6847-19659).

### Selecting the evaluation model

Multiple models exist in the literature for program evaluation [9], however, the purpose of evaluation facilitates selecting the most appropriate evaluation strategy. As explained by the complexity theory, many internal and external factors contribute to creating complex systems in medical education programs, for example, characteristics of the program participants, evolving knowledge of each discipline, varying professional practice, and the diverse environment in which the educational program function, etc. In light of this theory, the Context/Input/Process/Product (CIPP) process evaluation model was found to be the best fit for the formative evaluation of the fellowship programs at our institute [9].

### Creating the team of program evaluators

Six teams were created for evaluating all the paediatric fellowship programs. The selection of evaluators in each team was guided by the following criteria: each individual had to be a faculty member with at least five years of experience in academia and had to have previously held an academic leadership position. Each team comprised of four members i.e., two clinicians (paediatric specialists) with over ten-year service experience, one clinician-educator (paediatrician with formal training in health professions education), and a senior administrative support staff. The evaluators were not part of the faculty fellowship program being evaluated. A detailed briefing was given to the teams about the purpose of this evaluation ambiguities were removed. Each team was provided with some resource documents which are listed later in this text.

### Data collection methodology

The following resources were used for data collection.

1. Document review.

Each fellowship program has documents providing vital information about the program such as program curriculum, competency framework, program policies (including fellows’ recruitment, promotion, grievance), trainees’ job description, and academic performance record. Evaluating teams utilized the PGME Program Information Form (PIF) for document review. This form has two parts. The first explores the demographic details of the program, including but not limited to CPSP approval status, number of faculty on board and number of fellows enrolled in each sub-specialty, attrition/year, and number of graduates. The second part confirms if the documentary evidence is met (80–100%), partially met (50–75%), or not met (< 50%) for each source including training curriculum (including program outcomes, teaching and learning strategies, assessment, and feedback opportunities), clinical responsibilities and caseload. Compilation of this information leads to a quantitative account of each fellowship program. The decision regarding the scoring of the met, un-met, or partially met criteria was made after discussion among the reviewers.

2. Experiences of faculty members and fellows.

Open-ended question guides were developed by the department’s medical educationist to explore the experiences of both the trainees and trainers in each fellowship program. For the fellows, the discussion was based on their experiences of clinical and academic learning, challenges in training, the support system in the fellowship program, supervision, and suggestions for improvement. A separate meeting was kept with program faculty who shared their opinions about the program outcome, strengths, limitations/challenges, support system, and suggestions for improvement in the quality of the training program.

3. Observation of teaching session.

After obtaining permission from the individual programs, the reviewers observed one to two academic teaching sessions to gauge the learning environment, fellow’s contribution, faculty contribution, and innovation, if any, used in the session.

### Data analysis

The reports compiled by the individual review committees of all six paediatric fellowship programs were presented to the department’s fellowship committee. The collated data were broadly divided into two categories for the purpose of analysis and ease of reporting.

### Quantitative data

Data collected from the document review was analyzed using the Statistical Package for the Social Sciences (SPSS) version 22.0. Quantitative variables reported as percentages and frequency include, but are not limited to, the number of fellows in the training program, number of faculty in the training program, and percentage of documentary evidence met.

### Qualitative data

Reports of discussions with faculty and fellows and teaching session observation of each fellowship program were transcribed and underwent thematic analysis. Data were condensed into codes, which were clumped together to yield categories or subcategories. Similar categories were merged to extract themes by two independent reviewers to ensure trustworthiness.

## Results

### Quantitative results

The demographics of all the programs are shared in Table 1. Neonatology was the first fellowship program to start (1996) and subsequently receive training approval from CPSP (1998). The duration of most of the programs is two years except for Cardiology where CPSP mandates a three-year program. Neonatology has the greatest number of onboard fellows (n = 7) while Cardiology has the highest attrition rate (33%). Despite this, Cardiology trainees have a remarkable 71% passing rate for the CPSP exams. Notably, Padiatric Critical Care Medicine (PCCM) was found to have the most substantial research output.

**Table 1:** Basic Demographics

	Cardiology	Neonatology	Critical Care	Infectious Disease	Endocrinology	Neurology
**Year of commencement of Program**	2006	1996	2012	2006	2018	2010
Approval from CPSP*	2008	1998	2020	2012	2021	2011
**Duration of training (Years)**	3	2	2	2	2	2
**Current Number of fellows in the program/ Fellowship positions**	1/3	7/7	3/3	2/2	1/1	1/2
**Number of program alumni**	7	29	12	8	1	11
**Number of dropouts n (%)**	4 (33)	1 (3)	3(19)	1(7)	0 (0)	0 (0)
**Number of current faculty in each fellowship program**	5	5	4	6	2	4
**Faculty to Fellow Ratio**	5:1	5:7	4:3	2:1	2:1	4:1
Number of fellows who passed CPSP*certifying exam n (%)	5 (71.4)	9 (31)	First exam due in 2023	First exam due in 2022	First exam due in 2023	4 (36.3)
**Number of the fellow’s publications during fellowship training**	5	4	8	3	1	2

*College of Physicians and Surgeons Pakistan

The key findings are reported below according to the various domains of medical education.

### Curriculum

The curriculum is the reconstruction of knowledge and experience that enables the learner to grow in exercising intelligent control of subsequent knowledge and experience [16]. The study revealed structured curricula with well-defined objectives across all programs. The framework comprised six core competencies including medical knowledge, patient care, interprofessional communication skills, professionalism, system-based practice, and practice-based learning and improvement. Some programs have mandatory clinical rotations (duration: 1–2 months) as defined by CPSP, within and outside the institution, to enhance the scope of learning and enrich the trainees’ experiences. For example, infectious disease fellows rotate in community-based tuberculosis clinics in a tertiary care setup in Karachi. The curricula were also found to have supervision guidelines according to trainee level, details regarding teaching and learning strategies, core content, assessment strategies, and learning resources.

### Educational strategies

Educational Strategies provide the means by which the curriculum’s objectives are achieved. These are considered the heart of the curriculum [17]. During the review, it was noted that all six programs have the implementation of evidence-based learning highlighted via formal ongoing core sessions, case presentations, and bedside teaching. Faculty-led sessions from internal and external experts were also found to be included in the academic rosters. Additionally, intensive care fellowships, namely, neonatology and PCCM, were found to have simulation-based mock drills to train fellows on crisis resource management, interprofessional communication skills, teamwork, etc. (Table 2).

### Assessment and evaluation

Assessments are crucial in judging learners’ competencies, assisting their performance improvement through feedback, providing guidance, and aiding in trainee selection for advanced training [18]. All programs were found to have an online evaluation system of fellows by faculty through the one45 software, offering an automatic feedback mechanism after each rotation. Most of the programs have monthly/quarterly multiple-choice question assessments, aligned with the rotation/core topic. Workplace-based assessment (WPBA) entails the evaluation of daily clinical practices employed in the working situation [19]. Mini clinical evaluation exercise (Mini-Cex) and direct observation of procedural skills (DOPS) are the two most common WPBA used for formative assessment [20]. This has been incorporated by CPSP in all the newly approved fellowship programs, for example, PCCM (Table 2).

One of the gaps that were identified across all programs was an absence of opportunity for formal one-to-one feedback to fellows from program faculty (Table 3). This could be due to the close supervision of fellows in the program with opportunities of receiving informal feedback. Another aspect identified was the lack of summative assessment in all programs (Table 2). Summative assessment is crucial to outcome-based education as it provides the opportunity to document the level of competency attained by the fellow at the end of training [21]. One of the reasons could be the CPSP certification exam that takes place once the training requirement is completed.

**Table 2:** Educational and assessment opportunities in each program

Fellowship Program	Educational strategies	Formative Assessment	Summative Assessment
**Cardiology**	Morbidity and Mortality meetings, Core sessions, Case Conferences, Radiology, Bedside teaching	Monthly MCQs****, Continuous evaluation one45	None
**Neonatology**	Morbidity and Mortality meetings, Journal Club, Case Conferences, Fellow teaching sessions with residents, Faculty-led teaching sessions, Mock Drills, Bedside teaching	OSCE* Continuous evaluation one45	None
**Critical Care**	Patient care meetings, Tutorials, Case Conference, Journal Club, Faculty-led discussions, Protocol presentations, Bedside teaching	Mini-CEX**, DOPS***Monthly MCQS****, Continuous evaluation one45	None
**Infectious Disease**	Journal club, Core sessions, Case Conference, Microbiology, Bedside teaching	Monthly MCQS****, Continuous evaluation one45	None
**Endocrinology**	Core sessions, Tutorials, Case Conferences/Discussions, E-learning sessions (ESPE), Bedside teaching	Continuous evaluation one45	None
**Neurology**	Journal Club, Tutorials, Faculty talk, Neuroradiology sessions, Bedside teaching	OSCE* Continuous evaluation one45	None

*Objective Structured Clinical Examination

** Mini-clinical evaluation exercise

***Direct observation of procedural skills

**** Multiple choice questions

**Table 3:** Program Review; assessed on the bases of met (80–100%), partially met (50–75%), and not met (< 50%)

	Cardiology	Neonatology	Critical Care	Infectious Disease	Endocrinology	Neurology
Curriculum						
Not Met (< 50%)						
Partially Met (50–80%)						
Met (80–100%)	√	√	√	√	√	√
**Clinical training**						
Not Met (< 50%)						
Partially Met (50–80%)	√					
Met (80–100%)		√	√	√	√	√
**Evaluation and assessment**						
Not Met (< 50%)						
Partially Met (50–80%)	√	√	√	√	√	√
Met (80–100%)						
**Feedback (one: one)**						
Not Met (< 50%)		√	√	√	√	√
Partially Met (50–80%)	√					
Met (80–100%)						

### Qualitative results

Thematic analysis of the report of fellows and faculty discussion revealed three key themes as discussed below (Table 4).

**Table 4:** Content Analysis of the discussion report

Categories	Subthemes	Themes
- Curriculum consisting of broad academic, clinical, and research exposure; covering all aspects of training. - Supportive environment highlighting mentorship and teamwork. - Effluence of supportive skills including leadership, teaching, and presentation skills. - Strict continuous assessment to encourage sound clinical acumen.	- Holistic grooming of trainees. - Development of a skilled and stable workforce who are expert providers of care.	**Program aspects translating into strengthening the training**
**Categories**	**Subthemes**	**Themes**
- Significant lack of exposure to subspecialties, pathological diversity, research training, and hands-on practice. - Suboptimal feedback and weak evaluation. - Imbalance of workload vs. service provision. - Lacks standardization.	- Compromised learning of trainees. - Inability to provide continuing innovative graduate medical education.	**Gaps in the training program in delivering the expectations**
**Categories**	**Subthemes**	**Themes**
- Increasing resourcefulness of service providers. - Expanding exposure. - Improving learning opportunities. - Need for focused standardized evaluation.	- Establishing strong communication between trainers and trainees. - Tailoring the program according to the need of the trainees.	**Making ways to reach par excellence**

### Program aspects translating into strengthening the training

The fellowship programs exhibited a sound structure, encompassing a comprehensive curriculum of academic, clinical, and research aspects, as well as honing leadership and presentation skills. Academically, trainees appreciated diverse learning tools and resources that included expertise from national and international platforms. Opportunities for formative assessment laid out in the programs and were deemed as thorough, informative, and divided well throughout the duration of the rotation. Clinical teaching was found to be continuous, occurring during in-service rounds and clinics. The programs effectively integrated research and statistical skills, covering clinical and basic research along with biostatistics. Fellows had ample research opportunities under excellent mentorship. Non-clinical training, including leadership, teaching, and presentation skills, were embedded in all programs and well-received by trainees, who expressed interest in further expanding these opportunities. This holistic approach to training coupled with close mentorship offered by the faculty resulted in confident, efficient trainees and visibly improved patient care.

### Gaps in the training programs in delivering the expectations

Despite the structured curriculum, the need for clearer, standardized tools was recognized to ensure fellowship graduates meet all competencies. Identified areas for improvement included limited feedback, lack of summative assessment opportunities, and undefined aims for external/internal rotations. Fellows noted the absence of formal assessment during these rotations. WPBA was seen as underutilized in many programs, while the imbalance between service care providers and workload was considered an issue. Faculty noted that the diverse sociocultural backgrounds of fellows can extend the learning curve for system-based practices. They advocated for expanding fellows’ exposure to national and international healthcare systems through electives. However, financial constraints pose a significant challenge to this effort. Finally, while formal research training existed, a gap was observed in translating these skills into actual publications. The CPSP mandates that to qualify for the fellowship certifying examination fellows must author and submit a research paper linked to their specific training program. However, variations were observed in the practices related to research across the fellowship programs, encompassing aspects such as the provision of mentorship, formal learning opportunities regarding research, and the dedication of specific time slots within the programs for research activities.

### Making ways to reach par excellence

Group discussions highlighted potential improvements in training quality. While the patient load is substantial for learning, there is a compelling need to branch out to other institutes during rotations. Rotations in public sector institutes can assist in understanding the needs of people in underdeveloped areas who lack financial support to reach private institutes. Due to the strength of the research training being offered, it was voiced that a certain period may be set solely dedicated to it. It was also noted that assessment methods need to be more focused and aligned with the curricular objectives and outcomes for each training program. Faculty also expressed their opinion on the need for trainees to get administrative exposure and involvement in quality improvement projects.

## Discussion

Fellowship is intense training that follows certification in a primary specialty or subspecialty and focuses on distinct and advanced clinical and/or academic skills [5]. Objectives of a fellowship program can be broadly divided into clinical and non-clinical encompassing academic, clinical, and research responsibilities along with teaching, administrative, and leadership roles. Librizzi et al., compared competencies of fellowship-trained and non-fellowship-trained paediatric hospitalists, and found that fellowship-trained physicians felt more competent in managing patients with medical complexity, undertaking research projects, leading quality initiative programs, and educating trainees, as compared to non-fellowship-trained [22]. In a review of Pakistan’s post-graduate training, Bigg JS, an Australian educationist, found a staggering lack of exposure to subspecialty training. with no insight into a subspecialty career pathway was seen amongst trainees, even though the trainees’ confidence and skills were good, their study identified the need for more contextual and specialized experience [8]. In this report, we reviewed the six paediatric fellowship programs at AKU via process evaluation using the CIPP model.

Our results, showed that the curriculum was extensive with an appropriate competency framework however, lacked focused teaching, aligning with the trainees’ objectives. There is a need to set clear and standardized assessment tools to ensure that our fellowship graduates meet all competencies. Similar findings were reported in an extensive study done by Constance D. Baldwin on the strengthening of the academic base of general paediatrics fellowship programs where the main problem highlighted was the efficient integration of learning objectives in the curriculum [23]. In solution, available data should be reviewed and revised to develop a balanced and innovative curricular framework [1].

Clinical teaching is the highlight of a fellowship program. Most clinical application is found to be through experiential learning, which has been known to be the most effective way of medical learning [24]. A pressing need for structuring clinical teaching has been identified with the development of super sub-specialties exposure and emphasis on hands-on. Our study found utilization of mock codes blue in critical care specialty; however, simulation has been underutilized for the formal teaching of critical procedures, crisis resource management, etc. There is also a strong need for faculty development in simulation-based teaching. Allen et al. in a study carried out a simulation-based boot camp for paediatric cardiac fellows, in the result of which she found that 80% of trainees felt more prepared for clinical responsibilities with a significant increase in their confidence in all specific knowledge and skills related to the learning objectives [25]. Similarly, simulation-based training carried out for paediatric critical care fellows showed its role in improving both critical care knowledge to provide care and relationship management skills needed in teamwork [26]. Systems-based practice and practice-based learning should be a part of medical training and practice, during fellowship since they are imperative to convert competencies into a lifelong practice [16]. Therefore, it is important to create a program of didactic, hands-on/simulation-based activities, and self-guided learning opportunities. It is essential for faculty to constantly reassess the clinical landscape to make sure that trainees are receiving education along with gaining enough experience.

Research emerged as one of the strongest domains, by both the faculty and the fellows in our analysis It is crucial to building this skill as the fellowship experience expands a physician’s abilities to pursue hypothesis-driven scientific inquiry resulting in evolving contributions to the medical literature and patient care. Fellows were given adequate opportunities to develop mentored relationships and built on an infrastructure that promotes collaborative research. However, as identified by the Dana-Farber Cancer Institute in Boston for their paediatric hematology/oncology fellowship program, there is a need to encourage an individualized training program when it comes to research [25], as each fellow comes into training with different goals and experiences. The curriculum should allow maximum flexibility, as opposed to that highlighted along with exposure to all basic, clinical, translational, and laboratory research. In a tertiary setting, a fellow’s care of patients within any subspecialty is undertaken with appropriate faculty supervision and conditional independence. Faculty and alumni serve as role models of excellence, compassion, professionalism, and scholarship. They are a crucial component in career development, including professional development and career planning [27]. Furthermore, the positive outcomes, identified were the encouragement of teaching and presentation skills which were greatly appreciated by both the faculty and the fellows.

### Strengths and limitations

This study is the first of its kind in the region which reports the experience of the process evaluation of multiple paediatric fellowship training programs in a tertiary care center in a low-middle-income country. Methodical triangulation was utilized to explore the holistic quality of training programs which will facilitate the program evaluators to utilize this experience in national and international contexts. However, the scope of this study, conducted in a single private sector institute, presents certain limitations. The findings of our study may not be completely representative of other contexts, particularly programs in the public sector as well as other institutes operating under the umbrella of CPSP. As such, conducting a multi-center study would not only increase the generalizability of the results but enable greater understanding of the structure of fellowship programs across various settings and contexts. Thus, while acknowledging this limitation, it must also be recognized that this study is an avenue for future research to utilize the structured and robust mechanism of process evaluation we have proposed to present more comprehensive and contextually comparative evaluations of subspecialty fellowship programs.

## Conclusions

Evaluation of paediatric fellowship programs provided an opportunity to review not only the written curriculum but also informed the stakeholders about the strengths and gaps in the transfer of this curriculum. To ensure the optimum transfer of competencies, there is a need to create opportunities of formative as well as summative assessments in the program. The prospects include preparing these programs for accreditation by international bodies, for example, the Accreditation Council for Graduate Medical to enhance the quality and consistency of fellowships, increase their recognition, promote the academic vitality of future faculty, and make the discipline a more attractive career choice for outstanding residents.

## Data Availability

The datasets used and/or analyzed during the current study are available from the corresponding author on reasonable request.

## Declarations

### Authors’ information

All the authors belong to Department of Paediatrics and Child Health, Karachi, Pakistan.

## Abbreviations

CPSP: College of Physician and Surgeons Pakistan
AKU: The Aga Khan University
PGME: Postgraduate Medical Education
ERC: Ethical review committee
CIPP: Context/Input/Process/Product
PIF: Program Information Form
SPSS: Statistical Package for the Social Sciences
PCCM: Paediatric critical care medicine
WPBA: Workplace-based assessment
Mini-Cex: Mini clinical evaluation exercise
DOPS: Direct observation of procedural skills
OSCE: Objective structured clinical examination
MCQS: Multiple choice questions

## References

[1] Jerardi KE, Fisher E, Rassbach C, Maniscalco J, Blankenburg R, Chase L et al. Development of a Curricular Framework for Pediatric Hospital Medicine Fellowships. Pediatrics [Internet]. 2017 Jul 1 [cited 2023 May 16];140(1). Available from: https://pubmed.ncbi.nlm.nih.gov/28600448/. 10.1542/peds.2017-0698 28600448

[2] Shah NH, Rhim HJH, Maniscalco J, Wilson K, Rassbach C. The current state of pediatric hospital medicine fellowships: A survey of program directors. J Hosp Med [Internet]. 2016 May 1 [cited 2023 May 16];11(5):324–8. Available from: https://pubmed.ncbi.nlm.nih.gov/27042818/. 10.1002/jhm.2571 27042818

[3] Touma NJ, Siemens DR. Attitudes and experiences of residents in pursuit of postgraduate fellowships: A national survey of Canadian trainees. Canadian Urological Association Journal [Internet]. 2014 Nov 1 [cited 2023 May 16];8(11–12):437. Available from: http://www.pmc/articles/PMC4277525/. 10.5489/cuaj.2136 PMC4277525 25553159

[4] Bari A, Khan RA, Rathore AW. Postgraduate residents’ perception of the clinical learning environment; use of postgraduate hospital educational environment measure (PHEEM) in pakistani context. J Pak Med Assoc. 2018 Mar;68(3):417–22. 29540877

[5] Karpinski J, Ajjawi R, Moreau K. Fellowship training: a qualitative study of scope and purpose across one department of medicine. BMC Med Educ [Internet]. 2017 Nov 21 [cited 2023 May 16];17(1). Available from: https://pubmed.ncbi.nlm.nih.gov/29157228/. 10.1186/s12909-017-1062-5 PMC5697383 29157228

[6] Fitzgerald JEF, Giddings CEB, Khera G, Marron CD. Improving the future of surgical training and education: consensus recommendations from the Association of Surgeons in Training. Int J Surg [Internet]. 2012 Jan 1 [cited 2023 May 16];10(8):389–92. Available from: https://pubmed.ncbi.nlm.nih.gov/22449833/. 10.1016/j.ijsu.2012.03.012 22449833

[7] Ellis MC, Dhungel B, Weerasinghe R, Vetto JT, Deveney K. Trends in research time, fellowship training, and practice patterns among general surgery graduates. J Surg Educ [Internet]. 2011 Mar 16 [cited 2023 May 16];68(4):309–12. Available from: https://europepmc.org/article/med/21708369. 10.1016/j.jsurg.2011.01.008 21708369

[8] Biggs JSG. Postgraduate medical training in Pakistan: observations and recommendations. J Coll Physicians Surg Pak 2008 Jan;18(1):58–63. 18452674

[9] Frye AW, Hemmer PA. Program evaluation models and related theories: AMEE guide no. 67. Med Teach [Internet]. 2012 May [cited 2023 May 16];34(5). Available from: https://pubmed.ncbi.nlm.nih.gov/22515309/. 10.3109/0142159X.2012.668637 22515309

[10] Fishbain D, Danon YL, Nissanholz-Gannot R. Accreditation systems for Postgraduate Medical Education: a comparison of five countries. Adv Health Sci Educ Theory Pract [Internet]. 2019 Aug 1 [cited 2023 May 16];24(3):503–24. Available from: https://pubmed.ncbi.nlm.nih.gov/30915642/. 10.1007/s10459-019-09880-x 30915642

[11] Saaiq M, Khaleeq-Uz-Zaman. Residents’ perceptions of their working conditions during residency training at PIMS. J Coll Physicians Surg Pak. 2010 Jun;20(6):400–4. 20642971

[12] Norcini JJ. Indicators of the educational effectiveness of subspecialty training programs in internal medicine. Acad Med. 1995 Jun;70(6):512–6. 10.1097/00001888-199506000-00012 7786371

[13] Gearhart SL, Wang MH, Gilson MM, Chen B, Kern DE (2012). Teaching and assessing technical proficiency in surgical subspecialty fellowships. J Surg Educ.

[14] Cataldi ML, Kelly-Hedrick M, Nanavati J, Chisolm MS, Anne LW. Post-residency medical education fellowships: a scoping review. Med Educ Online. 2021 Dec;26(1):1920084. 10.1080/10872981.2021.1920084 PMC8118440 33970808

[15] Alam L, Khan J, Alam M, Faraid V, Ajmal F, Bahadur L. Residents’ perspective on the quality of postgraduate training programs in Pakistan - the good, the bad and the ugly. Pak J Med Sci [Internet]. 2021 Nov 1 [cited 2023 May 16];37(7). Available from: https://pubmed.ncbi.nlm.nih.gov/34912401/. 10.12669/pjms.37.7.4297 PMC8613050 34912401

[16] Beauchamp G, Beauchamp GA. “Curriculum Theory: Meaning, Development, and Use,” Theory into Practice, 21 (Winter, 1982), 23–27. CIRS: Curriculum Inquiry and Related Studies from Educational Research: A Searchable Bibliography of Selected Studies [Internet]. 1982 Jan 1 [cited 2023 May 16]; Available from: https://stars.library.ucf.edu/cirs/15.

[17] Thomas PA, Kern DE, Hughes MT, Chen BY. Curriculum development for medical education: A six-step approach [Internet]. Angewandte Chemie International Edition, 6(11), 951–952. Johns Hopkins University Press; 2015 [cited 2023 May 16]. 5–24 p. Available from: https://pure.johnshopkins.edu/en/publications/curriculum-development-for-medical-education-a-six-step-approach.

[18] Long S, Rodriguez C, St-Onge C, Tellier PP, Torabi N, Young M. Factors affecting perceived credibility of assessment in medical education: A scoping review. Adv Health Sci Educ Theory Pract [Internet]. 2022 Mar 1 [cited 2023 May 16];27(1):229–62. Available from: https://pubmed.ncbi.nlm.nih.gov/34570298/. 10.1007/s10459-021-10071-w 34570298

[19] Lockyer J, Carraccio C, Chan MK, Hart D, Smee S, Touchie C et al. Core principles of assessment in competency-based medical education. Med Teach [Internet]. 2017 Jun 3 [cited 2023 May 16];39(6):609–16. Available from: https://pubmed.ncbi.nlm.nih.gov/28598746/. 10.1080/0142159X.2017.1315082 28598746

[20] Norcini J, Burch V. Workplace-based assessment as an educational tool: AMEE Guide No. 31. Med Teach [Internet]. 2007 Nov [cited 2023 May 16];29(9):855–71. Available from: https://pubmed.ncbi.nlm.nih.gov/18158655/. 10.1080/01421590701775453 18158655

[21] Holmboe ES, Sherbino J, Long DM, Swing SR, Frank JR. The role of assessment in competency-based medical education. Med Teach [Internet]. 2010 Aug [cited 2023 May 16];32(8):676–82. Available from: https://pubmed.ncbi.nlm.nih.gov/20662580/. 10.3109/0142159X.2010.500704 20662580

[22] Librizzi J, Winer JC, Banach L, Davis A. Perceived core competency achievements of fellowship and non-fellowship-trained early career pediatric hospitalists. J Hosp Med [Internet]. 2015 Jun 1 [cited 2023 May 16];10(6):373–9. Available from: https://pubmed.ncbi.nlm.nih.gov/25755166/. 10.1002/jhm.2337 25755166

[23] Baldwin CD, Szilagyi PG, Dreyer BP, Bell LM, Baker RC, Cheng TL et al. Strengthening the academic base of general pediatrics fellowship programs: a national program and curriculum development project. Ambul Pediatr [Internet]. 2007 Sep [cited 2023 May 16];7(5):340–7. Available from: https://pubmed.ncbi.nlm.nih.gov/17870641/. 10.1016/j.ambp.2007.05.009 17870641

[24] Gupta M, Ringer S, Tess A, Hansen A, Zupancic J. Developing a quality and safety curriculum for fellows: lessons learned from a neonatology fellowship program. Acad Pediatr [Internet]. 2014 Jan [cited 2023 May 16];14(1):47–53. Available from: https://pubmed.ncbi.nlm.nih.gov/24126046/. 10.1016/j.acap.2013.06.009 24126046

[25] Allan CK, Tannous P, DeWitt E, Farias M, Mansfield L, Ronai C et al. A Pediatric Cardiology Fellowship Boot Camp improves trainee confidence. Cardiol Young [Internet]. 2016 Dec 1 [cited 2023 May 16];26(8):1514–21. Available from: https://pubmed.ncbi.nlm.nih.gov/28148335/. 10.1017/S1047951116002614 PMC7269113 28148335

[26] Henricksen JW, Troy L, Siefkes H. Pediatric Critical Care Medicine Fellowship Simulation Use Survey. Pediatr Crit Care Med [Internet]. 2020 [cited 2023 May 16];21(10):E908–14. Available from: https://pubmed.ncbi.nlm.nih.gov/32195908/. 10.1097/PCC.0000000000002343 32195908

[27] Williams DA, Porter ES, Lux IVSE, Grier HE, Mack JW, Orkin SH. Training program in cancer and blood diseases: Pediatric Hematology/Oncology Fellowship Program, Children’s Hospital Boston/Dana-Farber Cancer Institute. Am J Hematol [Internet]. 2010 [cited 2023 May 16];85(10):793–4. Available from: https://pubmed.ncbi.nlm.nih.gov/20730793/. 10.1002/ajh.21816 20730793

